# The Impact of Attention Mechanisms on Speech Emotion Recognition

**DOI:** 10.3390/s21227530

**Published:** 2021-11-12

**Authors:** Shouyan Chen, Mingyan Zhang, Xiaofen Yang, Zhijia Zhao, Tao Zou, Xinqi Sun

**Affiliations:** School of Mechanical and Electrical Engineering, Guangzhou University, Guangzhou 510006, China; maxcsy@gzhu.edu.cn (S.C.); 1807300151@e.gzhu.edu.cn (M.Z.); 1807700055@e.gzhu.edu.cn (X.Y.); tzou@gzhu.edu.cn (T.Z.); 2112007151@e.gzhu.edu.cn (X.S.)

**Keywords:** artificial intelligence, speech emotion recognition, attention mechanism, neural networks

## Abstract

Speech emotion recognition (SER) plays an important role in real-time applications of human-machine interaction. The Attention Mechanism is widely used to improve the performance of SER. However, the applicable rules of attention mechanism are not deeply discussed. This paper discussed the difference between Global-Attention and Self-Attention and explored their applicable rules to SER classification construction. The experimental results show that the Global-Attention can improve the accuracy of the sequential model, while the Self-Attention can improve the accuracy of the parallel model when conducting the model with the CNN and the LSTM. With this knowledge, a classifier (CNN-LSTM×2+Global-Attention model) for SER is proposed. The experiments result show that it could achieve an accuracy of 85.427% on the EMO-DB dataset.

## 1. Introduction

Speech Emotion Recognition (SER) has a wide range of potential applications in areas, such as human–robot interactions [1], computer-aided instruction, e-commerce, and medical assistance [2,3].

Traditional, effective analysis methods can be roughly divided into dictionary-based methods and machine-learning-based methods. The methods based on an emotion dictionary need to use an emotion dictionary which has been labeled manually. These methods rely heavily on the quality of the emotional dictionary, and the maintenance of the dictionary needs a lot of work and material resources. With the continuous emergence of new words, the dictionary cannot meet the application’s needs [4].

The machine-learning method has attracted great attention from researchers. Convolutional Neural Networks (CNNs) [3,5,6] and LSTM networks [7] and their combination [7,8,9] have been widely used. Misbah Farooq et al. [8] used a feature-selection algorithm based on CNN to reduce the influence of artificial design that was insufficient to accurately describe the emotional state of the speaker.

However, due to the mechanism of traditional CNN, traditional CNN is difficult to extract the high-dimensional speech features. In view of this, recurrent neural networks (RNN) is then proposed. Nevertheless, RNN takes sequence data as input and is capable to extract high-dimensional time series feature, RNN has performance degradation due to long-term dependence, gradient disappearance, and gradient explosion. LSTM is then proposed to address this issue, which can be combined with CNN has achieve a great success in SER. Jianfeng Zhao et al. [6] combines 1D and 2D CNN with LSTM and reaches an accuracy of 82.42% on the EMO-DB dataset. However, although CNN+LSTM can deal with time series well to a certain extent, it can’t achieve a satisfactory performance on feature extraction of length data. In view of this, Yawei Mu et al. [10] introduced Attention mechanism to establish CNN+BiLSTM+attention model and conducted experiments on the IEMOCAP data set, reaching the accuracy of 64.08%. Ranjana Dangol et al. [7] established the CNN+LSTM+Self-Attention model and achieved the accuracy of 83.38% on the EMO-DB data set. In addition, as mentioned by Dzmitry Bahdanau et al. [11], when the Attention mechanism and LSTM are used to construct models for speech recognition tasks, the principle of the attention mechanism is mainly to calculate the weight of core features in the LSTM framework with a single layer and extract information more relevant to the target. However, when there are two or more LSTM layers in the CNN-LSTM model, the impacts of Attention mechanism with different forms and positions are not clear, which is worth to further explore. This paper mainly explores the impacts of Attention mechanism with different forms and positions on LSTM, CNN, and CNNLSTM model. Three models are then established, which are CNN+LSTM×2+Global-Attention model, CNN+LSTM×2+Self-Attention model and CNN+LSTM+Global-Attention+LSTM model. By comparison, CNN+LSTM×2+Global-Attention model can achieve the accuracy of 85.427% on the EMO-DB dataset, which is better than the other two models. It is further reflected that the position and form of the attention mechanism have a certain influence on the model performance. By comparing the accuracy, convergence speed, and generalization ability of the model, we finally chose the CNN+LSTM×2+Global-Attention model to extract emotional features from miscellaneous voice information, and improved the recognition accuracy. The results of the exploratory experiment in this paper will provide some reference for subsequent researchers when using the attention mechanism.

Our major contributions in this article are documented below:

- **To explore the influence of difference Attention Mechanisms on different models:** We set up Sequential Networks and Parallel Networks respectively, to explore the influence of difference Attention Mechanisms on different models. As for the forms of the attention mechanism, we used Self-Attention and Global-Attention.
- **To propose a CNN-LSTM×2+Global-Attention model:** By comparing the training convergence speed, accuracy, and generalization ability of different models, we proposed a CNN-LSTM×2+global-attention model and conducted experiments on the EMO-DB dataset, which achieved an accuracy of 85.427%.

The paper is organized as follows: In Section 2, related studies are presented and discussed. Section 3 provides the architecture of the proposed models. Section 4 analyzes and discusses the experiments performed. Finally, conclusions are drawn in Section 5.

## 2. Related Work

Traditional methods of Speech Emotion Recognition (SER) are mainly based on basic acoustic emotion features and machine learning models. Since human emotions are complex and abstract, basic acoustic emotion features in traditional speech emotion recognition is not able to fully reflect emotional information, which limits the performance of machine learning models to a certain extent.

With the improvement of hardware and the growth of data volume, deep learning has rapidly made great progress and breakthroughs in various fields. With the powerful nonlinear representation capability of deep networks, speech emotion recognition has entered a new era. Zeiler et al. [12] visualized the features of Convolutional Neural Networks (CNN) through Transposed Convolution, showing that deep neural networks can combine low-level features into high-level semantic features. Human emotion is the knowledge of the high semantic category. It is an efficient and feasible method to extract speech emotion features through deep neural networks. Therefore, more and more researchers have tried to apply deep neural networks to SER and have achieved remarkable results. Zhu et al. [9] extracted deep emotion features from Mel-Frequency Cepstral Coefficients (MFCC), pitch, formants, short-term zero-cross rate, and short-term energy by using a combination model based on the Support Vector Machine (SVM) and Deep Belief Network (DBN). Zhong Qiu Wang et al. [13] used Deep Neural Networks (DNN) to encode the basic acoustic features of each audio segment into a fixed-length depth feature vector and identified emotions through Extreme Learning Machine (ELM). Zhao et al. [14] used the attention-based BiLSTM and the Fully Convolutional Networks (FCN) to learn spatiotemporal emotion features from the basic acoustic features, and then fed them to a machine-learning model for emotion classification. Kim et al. [14] extracted deep emotional features from 20 basic acoustic features through deep network EMNET (composed of CNN and LSTM). Some studies [10] have shown that different emotions show differences in speech frequencies, and there is more emotion-related information distribution between high and low frequencies. In recent years, researchers have attempted to replace basic acoustic emotional features with spectrograms. Different from the basic acoustic features, the spectrogram is a time–frequency diagram which can show how the energy of speech changes with time and frequency, and it can well reflect the time-domain and frequency-domain characteristics of speech while retaining temporal and local information. Therefore, the SER based on the combination of spectrograms and CNN has become a hot technology. Aharon Satt et al. [15] extracted deep emotional features from the spectrograms through CNN and LSTM. Nicholas Cummins et al. [16] also adopted spectrograms as input of pretrained CNN. Jinkyu Lee et al. [17] proposed the SER framework based on language spectrum and RNN-ELM. Lili Guo et al. [18] combined CNN depth features based on a spectrum and DNN depth features based on low-level acoustic features into a fixed-length feature vector to train ELM to perform SER. Although there has been a breakthrough in SER, how to effectively extract emotion features from lengthy speech has become a technical problem in the field of SER. Therefore, this paper adopts several existing neural network frameworks (CNN, RNN, LSTM, and the attention mechanism) to explore the role position of attention and the influence of its form on the model to provide some reference for subsequent researchers when using the attention mechanism. It can also extract emotional features from miscellaneous voice information and improve recognition accuracy.

In recent years, CNN has rapidly developed into a powerful technology and has achieved breakthrough achievements in many fields such as image, video, text, and voice. Therefore, CNN has also been applied in the field of SER by some researchers. Misbah Farooq et al. [8] used a feature selection algorithm based on CNN to reduce the influence of artificial design that was insufficient to accurately describe the emotional state of the speaker.

Traditional CNN are unable to directly acquire the correlation between present and former time series information. Therefore, Recurrent Neural Networks (RNN) is introduced. RNN, as a kind of neural network that takes sequence data as input, circulating in the evolution direction of the sequence and have all nodes linked in a chain, can learn the correlation of time series information. As mentioned by Pascanu et al. [19], the RNN contains a hidden layer, which constantly updates the output value with the change of time. Traditional RNN has performance degradation due to long-term dependence, gradient disappearance, and gradient explosion. Therefore, researchers proposed a variant of RNN called LSTM to improve it. LSTM solves these problems by using the concept of gating to selectively decide what information to be used.As an improvement of the RNN, the LSTM can solve the issue of gradient vanishing in RNN. Hochreiter et al. [20] described that the LSTM controls the storage and deletion of information in the network through a gating mechanism. However, although LSTM can improve the long-term dependency problem existing in RNN, it is hard to learn the information of a long time series. Therefore, Bahdanau et al. [11] proposed an attention mechanism to solve the above problems.

The attention mechanism is often used in speech recognition, object detection, and other tasks, and has demonstrated its powerful ability. For SER, the speech will be mixed with much information irrelevant to emotion, especially environmental noise and so on. The attention mechanism can better extract the emotional information of speech and remove the interference, which can improve the accuracy of the model. Ranjana Dangol et al. [7] proposed an emotion recognition system combining CNN and LSTM with a relationship awareness self-attention mechanism. The average recognition accuracy of this system can reach 81.05%. Minji Seo and Myungho Kim used visual attention CNN and visual word bags for cross-SER. They used Visual attention Convolutional Neural Network (VACNN) to pretrain the log-Mel spectra of the source dataset. Qianjin Du et al. [21] used the attention-based LSTM model and the 1D CNN model to extract and integrate the features in speech, respectively, and finally, input the features into the Softmax.

In the speech emotion-recognition task, the attention mechanism can be used to focus the model on the part that can better express the emotional information, in order to ignore the irrelevant information and to improve the recognition performance. However, there are many forms of attention. In this paper, a comparative experiment was conducted to contrast the effects of Self-Attention and Global-Attention on the model in the effective phonological task. At the same time, when the attention mechanism and LSTM are used to build models for speech-recognition tasks, the principle of the attention mechanism is mainly to calculate the weight of core features in the LSTM framework with a single layer [22], and extract information more relevant to the target. However, when there are two or more LSTM layers in the LSTM framework, it is worth further exploring how the position of the attention mechanism affects the model. Therefore, in order to explore the role of the attention mechanism and the influence of its form on the model, CNN+LSTM×2+Global-Attention model and CNN+LSTM×2+Self-Attention model CNN+LSTM+Global-Attention+LSTM model were established, respectively. The role of the attention mechanism and the influence of the form on the model were evaluated from the perspective of model accuracy, convergence rate, and model loss. The research conclusions of this paper can provide some reference for future researchers when using the attention mechanism. They can extract emotional features from miscellaneous voice information and improve recognition accuracy.

## 3. Proposed Method

### 3.1. Feature Introduction

The emotional feature used in our experiments is MFCC. The MFCC is one of the most widely used spectral-related features in SER, which can effectively describe the shape of the vocal tract, and the vocal tract shapes are different when different emotions are pronounced. Therefore, the model can distinguish different emotions from the MFCCs of speech. First, MFCCs are filtered by a set of filter banks in line with the frequency response characteristics of the human auditory system. This group of filters is called Mel-filters bank. In the frequency domain, this group of filters is dense in the low-frequency band (more filters) and sparse in the high-frequency band (fewer filters). The low-frequency amplitude–frequency response is large, while the high-frequency amplitude–frequency response is small. The human auditory system also conforms to such characteristics, being low-frequency sensitive and high-frequency insensitive.

The technology of MFCC extraction is to input the audio spectrum into a set of triangle filters (Meyer filter banks) to filter in the frequency domain according to the characteristics of human ears for sound frequency, where the relationship between the center frequency of the triangle filter and frequency *f* is shown in the Equation (1):

(1) $$\mathrm{Mel}\left(f\right)=2595\mathrm{lg}\left(1+\frac{f}{700}\right)$$

where f is the frequency in Hz.

### 3.2. Feature Extraction

The proposal of the MFCC is based on the concept of Mel frequency, which can compensate for the distortion of the convolutional channel and is one of the most commonly used and effective characterizations of parameters. The extraction process of MFCC includes the Normalization, the Framing, the Windowing, the Fast Fourier Transform (FFT), the Meyer-filter-bank Filtering, the Logarithmic Energy Calculation, and the Discrete Cosine Transform (DCT).

The extraction process is as follows: First, we traversed all WAV files of the EMO-DB database and saved the files’ path of all the voices. This was performed in order to use the method of path truncation to add labels to each file, and then read the WAV files and obtain the information on the WAV files, and finally extract the MFCCs of the WAV files. Among them, since only speech signals lasting over 250 ms contain enough information for emotion recognition, Hamming window with a length of 25 ms and sliding step length of 10 ms are used to carry out 64 STFT-Mel filtering processing. A total of 10 × 63 + 25 = 635 ms of fragments were processed. Since the sampling frequency in the experiment is 16 kHz, when 16 points are 1 ms and 25 ms corresponds to 400 points, the length of the Hamming window is 401. The process of adding Windows is shown in Equations (2) and (3), where (2) is the calculation of adding Windows, and (3) is the Window Function:

*S_W*(*n*) = *S*(*n*) × *w*(*n*) (2)

(3) $$w\left(n\right)=\{\begin{array}{cc} 0.5-0.5cos\left[2\pi n∕\left(n-1\right)\right]\; & 0\leq n=N-1\\ \;0 & \;other \end{array}\;$$

As the speech signal is continuous in the time domain, the features extracted only reflect the characteristics of the frame. In order to better reflect the time-domain continuity of the feature, we performed different treatments of MFCCs. Commonly used treatments are the first-order difference and the second-order difference. Let *c*(*t*) be the data point of the digital audio signal, and the difference calculation is shown in Equation (4):

(4) $$dt=\frac{\sum_{n=1}^{N}n\left(c_{t+n}-C_{t-n}\right)}{2\sum_{n=1}^{N}n^{2}}\;$$

### 3.3. Model Construction

In order to explore the impact of attention mechanism with different forms and positions on SER, several models are constructed. With the concern of article length, the model constuction process of one model is presented.

**Figure 1:**
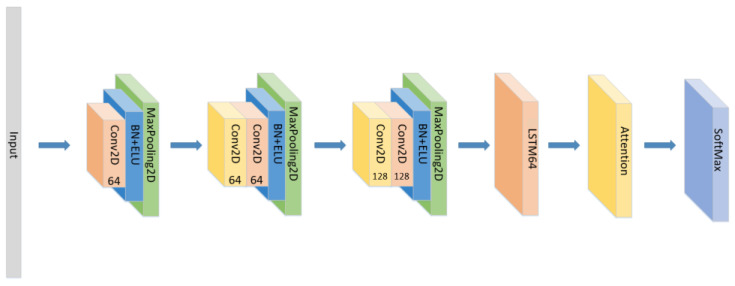
The detailed architecture of the proposed CNN+LSTM×2+attention model for speech emotion recognition.

A CNN-LSTM neural network is consists of 3 CNN blocks+LSTM+Attention block, which is shown in Figure 1.

**Figure 2:**
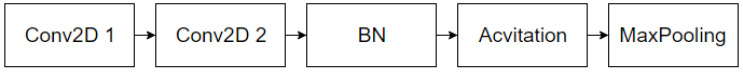
The overall architecture of the proposed CNN Blocks.

Among them, the CNN model has advantages in feature extraction. Its unique convolutional kernel can effectively extract local emotional features and obtain global emotional features at high levels. At the same time, the pooling operation can effectively adapt to different speech speeds and changes in speech positions to improve recognition accuracy. At the same time, CNN is based on local receptive fields and weight sharing. According to the rules, the CNN model with fewer parameters requires relatively less data in training. A deeper network is undoubtedly able to better extract high-dimensional emotional features. Therefore, the CNN model can extract speech features with better emotional description ability in the case of less data volume. It is undoubtedly a good feature extractor. So, for the CNN block, its structure is shown in the Figure 2. Since there are only 535 voice files in the EMO-DB dataset, and only MFCC and its first-order and second-order differentials are used as the input layer, only three CNN blocks are used in this paper to prevent overfitting caused by excessive computation and too complex of a network.

However, speech emotion recognition is based on speech sequence, and the CNN model cannot make good use of the time information in it, so it has some limitations. Therefore, the LSTM network, a variant of RNN, is added after the CNN blocks in this paper. Through a gating mechanism, LSTM controls the storage and deletion of information in the network. Each LSTM contains a hidden layer and a memory layer that controls network status updates and output values. The gates used in LSTM include the output gate, input gate, and forgetting gate. The output information $\tilde{c}$ is controlled by the parameters of each gate. $x_{t}$ and $h_{t}$ respectively represent the input value and output value of the LSTM network.

Candidate memory unit information $\tilde{c}$ at time t is calculated as the Equation (5). The input gate $i_{t}$ is determined by the current input data $x_{t}$ and the previous time unit output $h_{t-1}$, as in Equation (6). The forgetting gate $f_{t}$ controls the transmission of historical information, as per Equation (7). The output gate $O_{t}$ calculates the output value $h_{t}$ of the LSTM unit, as per Equation (8).

(5) $$\tilde{c}=tanh\left(W_{xc}x_{t}+W_{h_{c}}h_{t-1}+b_{c}\right)\;$$

(6) $$i_{t}=\sigma \left(W_{xi}x_{t}+W_{h_{i}}h_{t-1}+b_{i}\right)\;$$

(7) $$f_{t}=\sigma \left(W_{xf}x_{t}+W_{h_{f}}h_{t-1}+b_{f}\right)\;$$

(8) $$O_{t}=\sigma \left(W_{xo}x_{t}+W_{h_{o}}h_{t-1}+b_{o}\right)\;$$

where *σ* is the logical Sigmoid function and *W* is the weight.

By taking the output value of each independent hidden layer as the input value of the next hidden layer, the RNN with multiple hidden layers can be built, and even the deeper RNN with a more complex structure can be built.

For speech recognition, LSTM is generally the first choice. It can fit the data well based on time, and calculate outputs by memorizing information, which has achieved good results in various tasks related to time series. However, according to the structure of LSTM, a hidden state will be obtained at every moment *s*, which is called $h_{s}$, and the target hidden state will be obtained at the last moment *t*, which is called $h_{t}$. In the traditional LSTM application process, $h_{s}$ is often not used. Therefore, there will be a problem: LSTM has the forgetting gate $f_{t}$, which will forget the speech information that enters the network to a greater extent, so the network is more inclined to remember the later speech information, that is to say, the output $h_{t}$ will pay too more attention to the information at the end of the speech. Therefore, the attention layer is introduced.

**Global-Attention**

First, $a_{t}\left(s\right)$ is used to represent the weight of the hidden state at the moment s in all the hidden states. The weight of the hidden state at all times t is combined to obtain $a_{t}$, whose length is consistent with the time step. As for the weight $a_{t}\left(s\right)$ in a speech at the moment *s*, it is determined by the $h_{s}$ at the time *s* and the $h_{t}$ of the whole speech:

(9) $$a_{t}\left(s\right)=align\left(h_{t},h_{s}\right)=\frac{exp\left(score\left(h_{t},h_{s}\right)\right)}{\Sigma_{s^{\prime }}exp\left(score\left(h_{t},h_{s^{\prime }}\right)\right)}\;$$

where the score function is:

(10) $$score(h_{t},h_{s})\;=\;\{\begin{array}{cc} h_{t}^{T}\overset{—}{h_{s}} & \;\;dot\\ h_{t}^{T}W_{a}\overset{—}{h_{s}} & general\\ v_{a}^{T}tanh(W_{a}[h_{t},\overset{—}{h_{s}}]) & concat \end{array}$$

In order to accelerate the calculation speed and reduce the complexity of the model, the *dot* model is chosen.

Firstly, the two quantities in LSTM, the $h_{s}$ and the $h_{t}$*,* and the score at each moment through the score function are obtained. Then the Softmax function was used to normalize the scores of all moments to get the weight of each moment $a_{t}\left(s\right)$. By multiplying $a_{t}\left(s\right)$ and $h_{s}$, we can calculate the correlation vector $C_{t}\left(s\right)$ before and after the Global-Attention. $C_{t}$ of the whole speech segment can be obtained after averaging or summation of $C_{t}\left(s\right)$ at each moment, which can be considered as the vector of the speech segment.

**Self-Attention**

For the self-attention, the $h_{t}$ is first transmitted to a fully-connected layer. The resulting output $u_{\left(t\right)}$ is used for comparison with a trainable parameter matrix u (random initialization), used to represent context information to obtain $a_{t}\left(s\right)$ (alignment coefficient). Then, Softmax is used for normalization, and the specific calculation is shown in the following Equations (11)–(13):

(11) $$u_{\left(t\right)}=tanh\left(Wh_{t}\right)\;$$

(12) $$a_{t}\left(s\right)=align\left(h_{t},h_{s}\right)=\frac{exp\left(score\left(u_{\left(t\right)},u\right)\right)}{\Sigma_{s^{\prime }}exp\left(score\left(u_{\left(t\right)},u\right)\right)}\;$$

(13) $$s=\Sigma a_{t}\left(s\right)h_{t}\;$$

where s is the final output vector.

It can be concluded from the above formula that the principle of the attention mechanism is mainly to calculate the core weight of features. There is no decoder module in self-attention, and the distribution coefficient matrix in it has indicated the association between words in the context. The context vector $C_{t}\left(s\right)$ in the global-attention model represents the association between the words in the source sentence and the target words to be generated.

**Figure 3:**
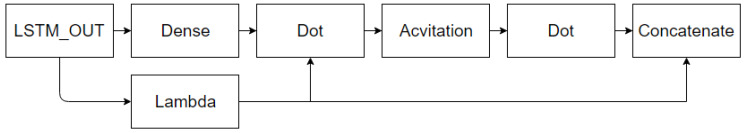
The overall architecture of the proposed global attention.

**Figure 4:**
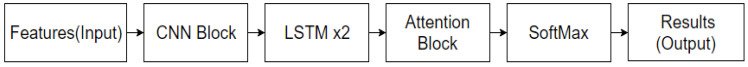
The overall process from input to output.

Therefore, the form and position of the attention mechanism will affect the calculation of weights and ultimately affect the performance of the model. In simply stacking LSTM layers, if the attention mechanism is set between two LSTM layers, the attention mechanism can only act on the features extracted by the first LSTM layer. The attention block is embodied in the structure of the web, as shown in Figure 3 below:

So, based on the advantages and disadvantages of CNN, RNN, and the effectiveness of the attention mechanism, we used the CNN as the emotional-feature extraction apparatus and the RNN as a classifier for emotion recognition in the model. We combined the advantages of CNN’s feature extraction with the LSTM gating mechanism and the attention mechanism to improve the recognition accuracy. The model structure is shown in Figure 4 below:

By calculating MFCC and its first-order and second-order differentials, and by stacking them together, a three-channel color picture (64 × 64 × 3) with transverse length related to signal duration and longitudinal length related to filter bank, is obtained, which is the input layer of the network. The Python speech-feature built-in functions were used to extract the above three features. For network parameter settings, see Table 1:

This is an improvement on the negative part of RELU. When *x* is less than zero, the ELU activation function adopts a method similar to the exponential calculation.

**Figure 5:**
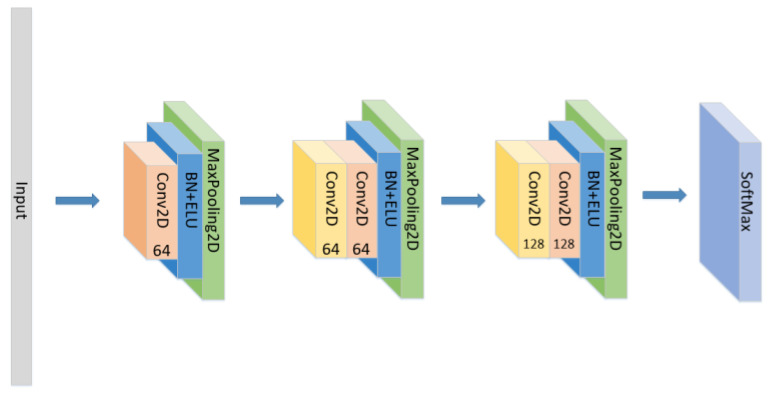
The construction of the CNN model.

**Figure 6:**
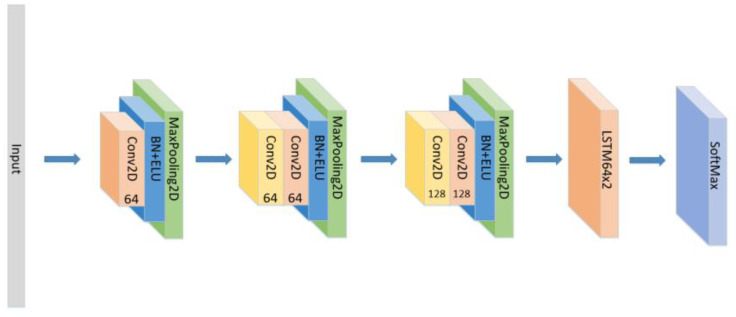
The construction of the CNN+LSTM×2 model.

**Figure 7:**
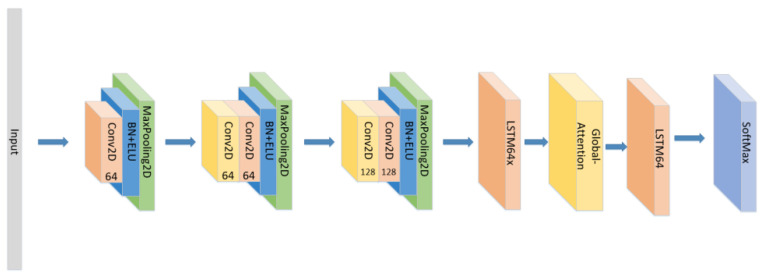
The construction of the CNN+LSTM+Global-Attention+LSTM model.

**Figure 8:**
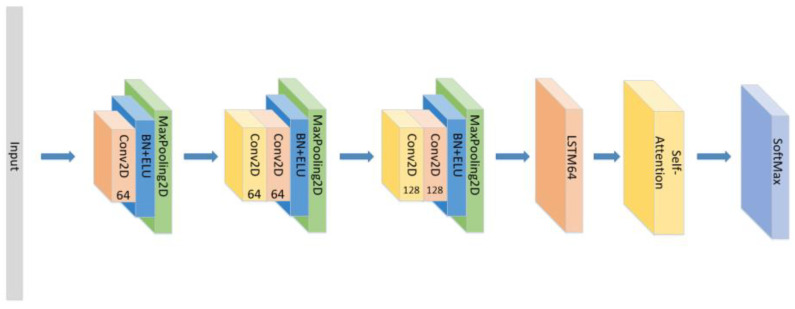
The construction of the CNN+LSTM×2+Self-Attention model.

**Figure 9:**
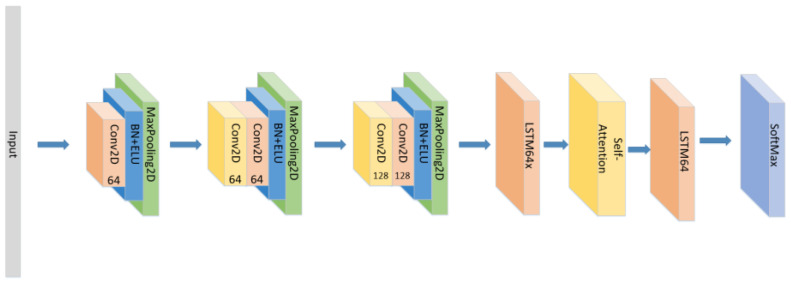
The construction of the CNN+LSTM+Self-Attention+LSTM model.

**Figure 10:**
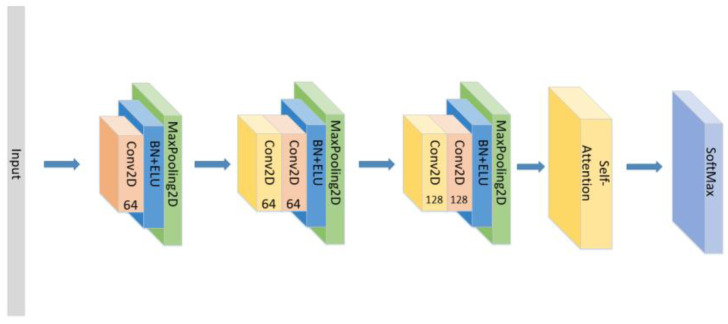
The construction of the CNN+Self-Attention model.

**Figure 11:**
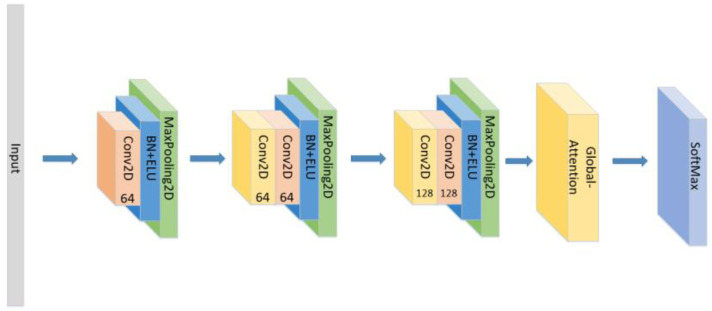
The construction of the CNN+Global-Attention model.

In addition to the model proposed above, we also conducted the following comparative experiments:

(1) Set the CNN network with the same parameters and the same number of layers as the original model without the LSTM layer and attention block as seen in Figure 5, and compare the experimental results.
(2) Set the CNN network with the same parameters and the same number of layers as the original model, add the LSTM layer as seen in Figure 6 and compare the experimental results without using the attention block.
(3) The position of the attention mechanism was changed as seen in Figure 7, and the CNN+LSTM+Global-Attention+LSTM network with the same parameters as the original model was set to explore the influence of the position of attention on the accuracy of the model.
(4) Change the form of the attention mechanism as seen in Figure 8 and set the CNN+ LSTM×2+Self-Attention network with the same parameters as the original model to explore the influence of the form of attention on the accuracy of the model.
(5) The position of the attention mechanism was changed as seen in Figure 9, and the CNN+LSTM+Self-Attention+LSTM network with the same parameters as the original model was set to explore the influence of the form of attention on the accuracy of the model.
(6) Set the CNN network with the same parameters and the same number of layers as the original model with the Self-Attention as shown in Figure 10.
(7) Set the CNN network with the same parameters and the same number of layers as the original model with the Self-Attention as shown in Figure 11.

## 4. Experiments and Results

### 4.1. Data Processing

The dataset used in this experiment is the EMO-DB dataset [23]. The EMO-DB dataset is a German emotional speech database recorded by the Berlin Institute of Technology. Five men and five women simulated seven emotions (neutral, anger, fear, happy, sadness, disgust, boredom) in 10 sentences (5 long and 5 short). The dataset contains 800 sentences, with a sampling rate of 48 KHz (compressed to 16 KHz) and 16-bit quantization. Through the hearing experiment of 20 participants (10 men and 10 women), 84.3% hearing recognition rate was obtained. This dataset retained 233 male emotional sentences and 302 female emotional sentences, for a total of 535 sentences. After MFCC extraction, the final voice signal size is a 64 × 64 × 3 array. Finally, an array of 3745 × 64 × 64 × 3 containing all MFCCs of voices (3745 is the total number of speech data points) can be obtained. The training data and test data were obtained by dividing them proportionally.

In this experiment, voice-sample labels were vectorized and converted into one-hot coding. This is essentially a binary vector, with only the index bit being 1 and the rest being 0. Labels used in this experiment and their corresponding relations are shown in Table 2:

In addition, the extracted samples were randomly scrambled, and the training set and test set were divided in a ratio of 8:2. Since it is difficult to guarantee the equal length of the difference between the recording time of the speech samples, it is necessary to normalize the speech length before extracting the features, this is to conduct zero-filling processing for the samples shorter than the threshold value, and truncate the samples higher than a threshold value.

### 4.2. Model Fitting

When compiling the network model, we used the SGD optimizer, and the learning rate was set to 0.0001. The loss function adopted is the Categorical_Crossentropy function because the label is vectorized by one-hot coding during the dataset partition and sorting. In order to find the approximate accuracy range, we set up 1000 epochs. The results show that after training 250 epochs, the changing of the loss value and accuracy are slow. Finally, we obtained the curve of the loss value and accuracy.

### 4.3. Experimental Environment Configuration

The computer for this experiment has the following parameters: the CPU is Intel Core i5-10030H 2.5 GHz (the Intel, Santa Clara, CA, USA), and the IDE uses Python 3.5 on Win10 (64-bit) system (Microsoft, Redmond, WA, USA), TensorFlow 2.2.0, and Keras 2.2.1 (the Google, Santa Clara, CA, USA).

### 4.4. Experimental Results

**Figure 12:**
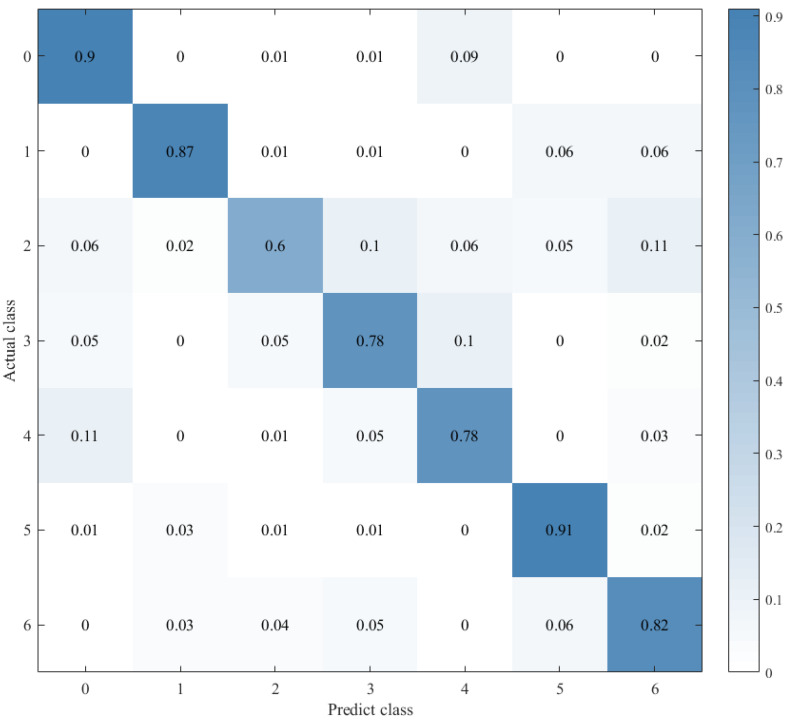
Confusion matrix for CNN+LSTM×2+Global-Attention.

**Figure 13:**
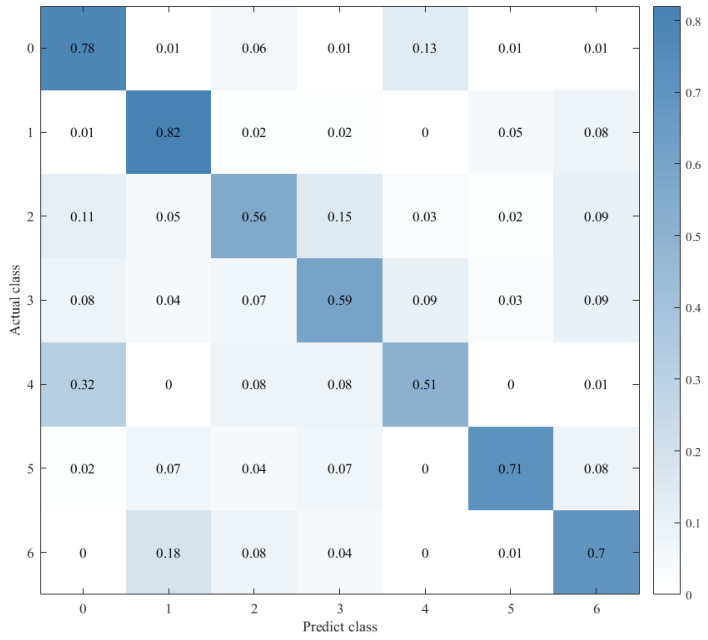
Confusion matrix for CNN.

**Figure 14:**
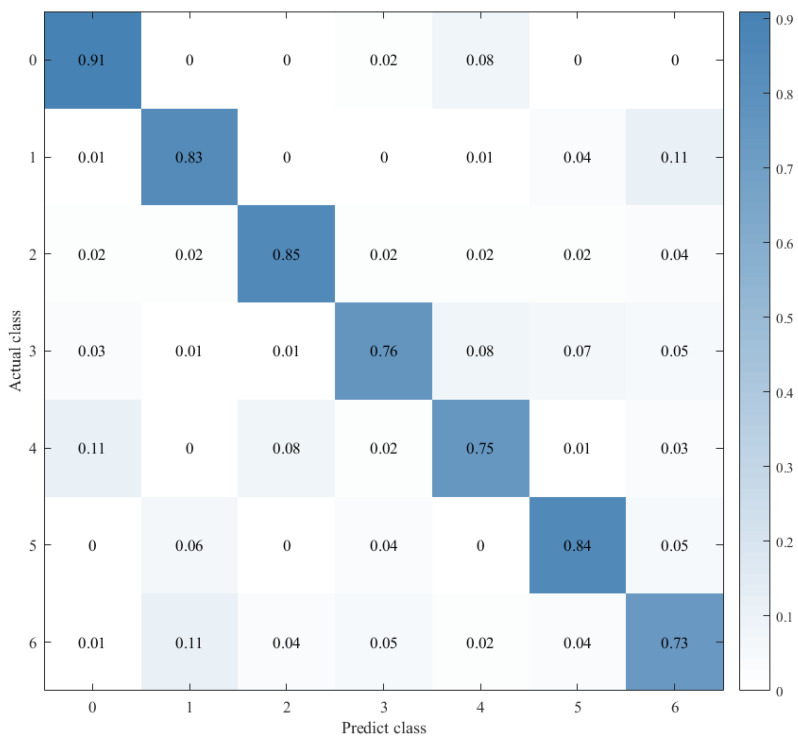
Confusion matrix for CNN+LSTM×2.

Combined with the results of the same number of epochs between CNN alone and CNN+ LSTM alone, and combined with the comparison of the confusion matrix as shown in Figure 12, Figure 13 and Figure 14, we found that the prediction accuracy of the CNN+LSTM×2+Global-Attention model is significantly better than that of the CNN network alone and the CNN+ LSTM×2 method alone. Moreover, the accuracy of the test data of CNN+LSTM×2+Global-Attention can reach 85.427%.

Combined with the results of CNN alone and CNN+LSTM2 alone after the same number of iterations and combined with the confusion matrix comparison, which was showed in Figure 13 and Figure 14, the test-set accuracy of the CNN+LSTM×2 model is better than that of the CNN model, that is, the former has stronger generalization ability than the latter. This proves again that although the CNN model can perform feature extraction, it can be used in speech emotion-recognition tasks, but the recognition accuracy is relatively low. Based on CNN, LSTM is added to learn the correlation of speech sequence time through LSTM, which helps to improve the accuracy of speech emotion recognition.

On this basis, the attention mechanism was introduced, and the CNN+LSTM×2+ Global-Attention model was established. This model is significantly superior to the CNN network alone and the CNN+LSTM method alone in terms of the accuracy of the test set, and the accuracy can reach 85.427%, which is shown in Figure 12**.** This result also proves again that, based on CNN+LSTM, the introduction of the attention mechanism to calculate the core weight of features is helpful to further extract the emotion-related information and ultimately improve the accuracy of the model. However, when there are two or more LSTM layers in the LSTM framework, we assume that the features extracted through the first LSTM layer are called local features. The features extracted through the second LSTM layer on this basis are called global features. The principle of the attention mechanism is to calculate the weight of core features. When there are two LSTM layers in the LSTM framework, the position of the attention mechanism will affect the feature objects it functions on and ultimately affects the performance of the model.

**Figure 15:**
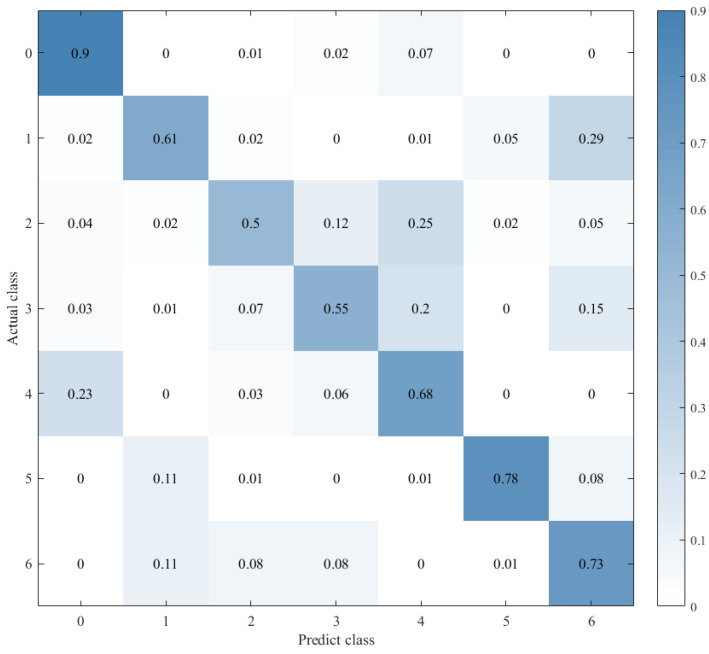
Confusion matrix for CNN+LSTM+Global-Attention+LSTM.

**Figure 16:**
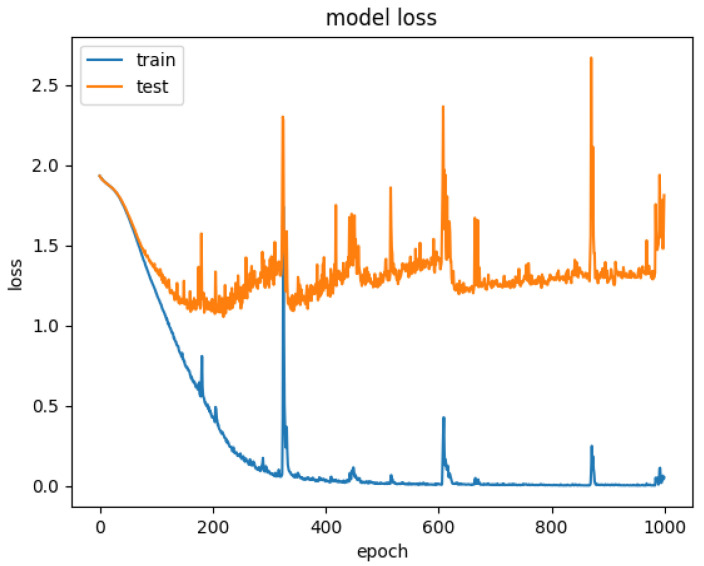
Model loss for CNN+LSTM+Global-Attention+LSTM.

**Figure 17:**
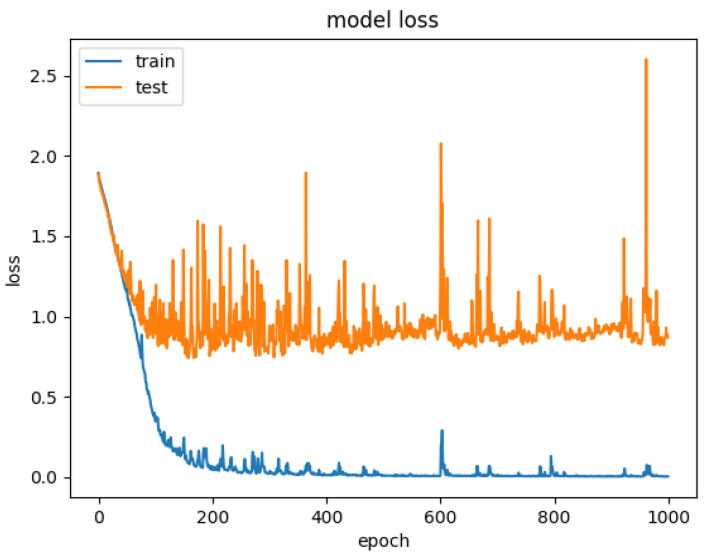
Model loss for CNN+LSTM×2+Global-Attention.

Therefore, to explore the effect of the position of attention on the model performance, we also conducted comparative experiments to establish the CNN+LSTM×2+Global-Attention model and CNN+LSTM+Global-Atention+LSTM model, respectively. By comparing the confusion matrices of the two models, which were shown in Figure 12 and Figure 15, it can be concluded that the accuracy of the attention mechanism is better when the object of attention is a global feature. By comparing the LOSS curves of the two models, which were shown in Figure 16 and Figure 17, when the attention mechanism acted on the global feature, the convergence rate of the model was faster and the model LOSS value of the test set was lower than the train set. On the contrary, when the attention mechanism acted on local features, the convergence rate of the model was relatively slow, and it can be seen from the LOSS curve that the LOSS value of the model showed a slight upward trend in the later period of training, indicating that there was a certain overfitting of the model.

**Figure 18:**
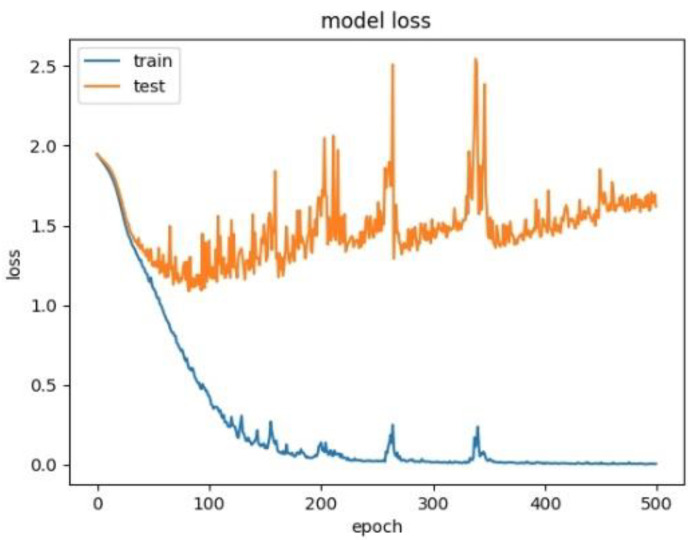
Model loss for CNN+LSTM×2+Self-Attention.

In order to explore the influence of the form of attention on the performance of the model, we established the CNN+LSTM×2+Self-Attention model. We compared it with the CNN+LSTM×2+Global-Attention model and the convergence rate of the two models was not much different, which was shown in Figure 17 and Figure 18, but the accuracy of the former model was low. Moreover, after the convergence point of the former is passed, the oscillating range of the LOSS value is relatively large, which suggests that models with self-attention have a greater tendency to overfitting. The experiments results are shown in Table 3.

**Figure 19:**
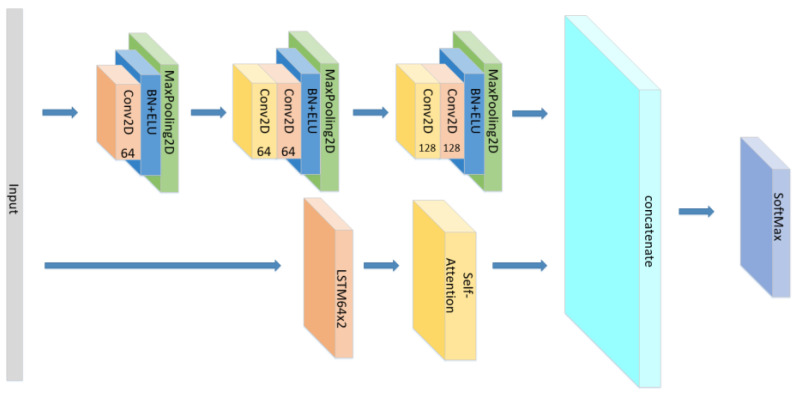
The structure of the CNN//LSTM×2+Self-Attention.

**Figure 20:**
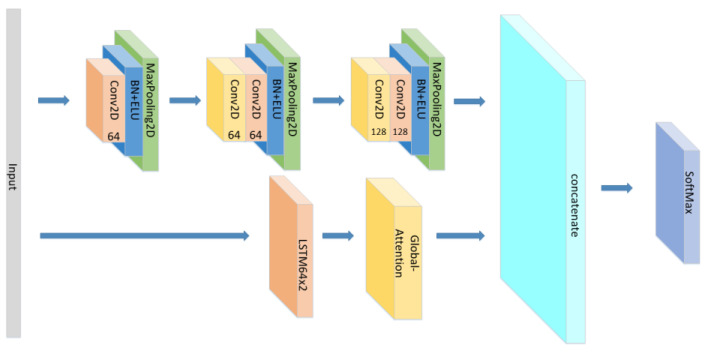
The structure of the CNN//LSTM×2+Global-Attention.

**Figure 21:**
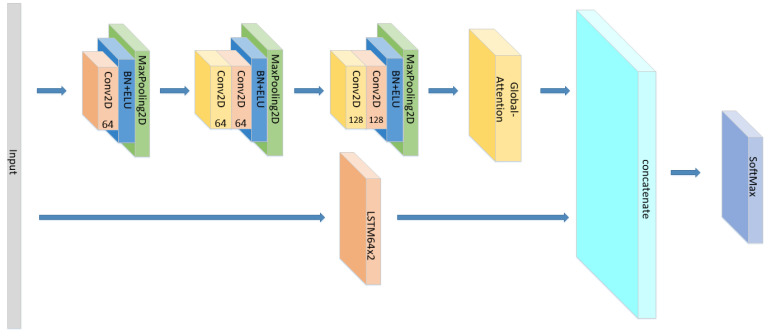
The structure of the CNN+Global-Attention//LSTM×2.

**Figure 22:**
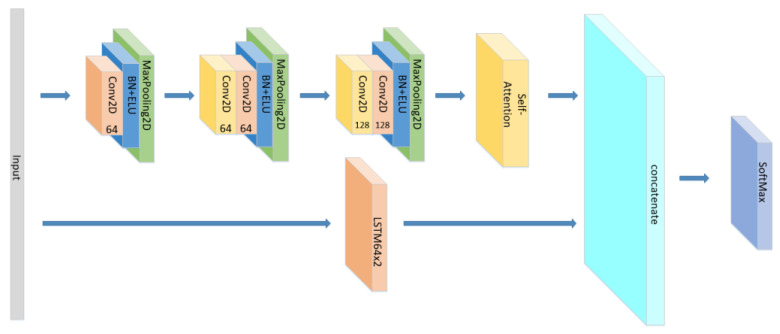
The structure of the CNN+Self-Attention//LSTM×2.

Since we only discussed the impact of Attention mechanism on the sequential network previously, we could not better explain the influence of different Attention mechanisms on different models. Therefore, we added extra experiments in the Table 3. The difference between parallel network and sequential network is that parallel network is a side-by-side structure of different types of networks. The sequential network was originally proposed in this paper to connect different networks in sequence, such as Input+CNN+LSTM+Attention+Output. In order to directly display the structure of parallel network, we made schematic diagrams according to the model we add, as Figure 19, Figure 20, Figure 21 and Figure 22. Each branch has its own input layer, which can be the same or different. During training, the branches do not affect each other. Since we need comparative experiments in this paper, the same input layer is selected for each branch. Finally, the features extracted from different branches are concatenated to obtain an output result.

Our intention is to explore the impact of the different Attention mechanisms on different networks. In the sequential network, the Attention mechanism considers the features extracted by LSTM when calculating the weight. The sequential network is a network whose structure is conducted by different networks in turn, which means that the input of the LSTM is related to the output of the CNN. This may cause interference between different networks in the sequential network. Therefore, it is impossible to further understand the influence of Attention mechanism in sequential network when it acts on one network alone. Furthermore, parallel network also has the advantage of introducing different network structures into branches and extracting features from their respective perspectives, which can give full play to the advantages of each network. Therefore, it is necessary to set up parallel networks.

We set up group 1 mainly to explore the influence of the forms of Attention mechanism on the model when it acts on LSTM alone in the parallel network. We set up group 2 mainly to explore the influence of the forms of Attention mechanism on the model when it acts on CNN alone. According to the group 1 and 2, it can also be discussed that the formal effects of different Attention mechanisms acting independently on different networks in parallel networks. We set up group 3, mainly exploring whether CNN and LSTM based on which all experiments in this paper are correct. We set group 4 and group 5 mainly to explore the influence of different Attention mechanisms on different sequential networks. Comparing the results of the parallel networks and the sequential networks, we found a special phenomenon. The ability of the Self-Attention to improve the accuracy of CNN+LSTM in sequential networks is worse than that of Global-Attention. However, the opposite is apparent in parallel networks.

As shown in group 1 and group 2, regarding to a parallel network which is constructed by CNN and LSTM×2, Self-Attention has a better performance than global attention. However, the opposite result is shown in group 5. Regarding a sequence network which is constructed by CNN and LSTM, global attention achieves a good accuracy at 85%, while self-attention only has 78.7%. This may be due to the difference in the time steps’ attention weights between self-attention and global attention, while the time steps scale of self-attention is smaller than that of global-attention. In the Sequential Network, self-attention is more suitable for CNN. This shows that the attention mechanism can be applied to the CNN network. The attention mechanism works better for the Sequential Network, as shown by the accuracy of groups 1, 2 and 5. This is because the features extracted by CNN and LSTM can be fully taken into account when calculating the weight of attention in the Sequential Network. However, in the Sequential Network, Global-Attention works better than Self-Attention. Self-Attention is only a mechanism for several elements inside of the input or output, which means that some important elements may be ignored, weakening the connection between the input and the output in Sequential Network. However, the Global-Attention mechanism would consider all elements of the input, and it is a mechanism occurring between the input and the output. According to the accuracy of group 5 and 6, attention is more suitable for the Sequential Network after the LSTM Network. This is partly due to the data structure, but the larger reason is that attention weights are calculated based on LSTM.

In addition, the method proposed in this paper was compared with the methods proposed by others on the same dataset, as shown in the table below in Table 4. Using the same dataset, Ranjana Dangol et al. [7] conducted a CNN- and attention-based LSTM model with an accuracy of 83.38%. They used the self-attention mechanism in their model. Abdul Malik Badshah et al. [24] built a simple CNN model and achieved an accuracy of 73.57%. Kai Zheng et al. [3] conducted a model of multilevel residual CNN and achieved an accuracy of 74.36%. As the CNN model only extracts some spatial features, the model accuracy cannot be further improved if only CNN is used to build the model above. Jianfeng Zhao et al. [6] used a 2DCNN+LSTM method and achieved an accuracy of 82.42%. It is clear that the structure of the CNN+LSTM can improve the accuracy of the model to a certain degree. Mingyi Chen et al. [25] used a 3D ACRNN model and achieved an accuracy of 82.82%. Global attention was used in the model. After comparison, we also fully considered the structural form of CNN+LSTM+attention, but we modified the number of CNN layers and LSTM layers used. In this way, the complexity of the model can be reduced, the accuracy of the model can be improved and overfitting can be avoided. Finally, experiments show that the accuracy of the proposed model is superior to the above model.

## 5. Conclusions

In this paper, we discussed the difference between Global-Attention and Self-Attention and explored their applicable rules to SER classification construction. First, we extracted the MFCC from the speech dataset as features and used it and its first-order and second-order derivatives as the input layer of the model conducted in this paper. By comparing the CNN model with the CNN+LSTM model, the accuracy of CNN+LSTM is shown to be significantly better than that of CNN alone, indicating that the LSTM model can learn the continuity between speech sequences and plays a good role in processing information about time series. To explore the impact of the Attention mechanism on the sequential networks, we conducted the construction of the CNN+LSTM+Attention and set up experiments on the EMO-DB dataset. Besides, to further discuss the influence of the Attention Mechanism on the SER, we also conducted parallel networks. The experimental results show that the Global-Attention can improve the accuracy of the sequential model, while the Self-Attention can improve the accuracy of the parallel model when conducting the model with the CNN and the LSTM. By comparing the results of all models, we proposed a CNN+LSTM×2+Global-Attention model for speech emotion recognition. The experiment result show that it can achieve an accuracy of 85.427% on the EMO-DB dataset. By comparing the model proposed in this paper with the methods of others, the CNN+LSTM×2+Global-Attention model proposed in this paper has certain advantages in the task of speech emotion recognition. In addition, the research results of this paper will provide some references for researchers to use attention for speech recognition tasks.

## Figures and Tables

**Table 1:** The network parameter Settings.

	Layers	Size	Strides	Output Shape	Param
Input shape				(64,64,3)	0
CNN BLOCK1	Conv2D(64)	(3,3)	(1,1)	(64,64,64)	1792
	BN				256
	Activation(elu)				0
	Maxpooling2D	(2,2)	(2,2)	(32,32,64)	0
CNN BLOCK2	Conv2D(64)	(3,3)	(1,1)	(32,32,64)	36,928
	Conv2D(64)	(3,3)	(1,1)	(32,32,64)	36,928
	BN			(32,32,64)	256
	Activation(elu)			(32,32,64)	0
	Maxpooling2D	(4,4)	(2,2)	(15,15,64)	0
CNN BLOCK3	Conv2D(128)	(3,3)	(1,1)	(15,15,128)	73,856
	Conv2D(128)	(3,3)	(1,1)	(15,15,128)	147,584
	BN			(15,15,128)	512
	Activation(elu)			(15,15,128)	0
	Maxpooling2D	(4,4)	(2,2)	(6,6,128)	0
LSTM layers	LSTM(64)			(36,64)	49,408
	LSTM(64)			(36,64)	33,024
attention block				(0,64)	4096
SoftMax	Dense(7)			(0,7)	455

**Table 2:** Labels used in this experiment and their corresponding relations.

Emotion	Label	Number	One-Hot Coding
anger	W	0	[1 0 0 0 0 0 0]
boredom	L	1	[0 1 0 0 0 0 0]
disgust	E	2	[0 0 1 0 0 0 0]
anxiety/fear	A	3	[0 0 0 1 0 0 0]
happiness	F	4	[0 0 0 0 1 0 0]
sadness	T	5	[0 0 0 0 0 1 0]
neutral version	N	6	[0 0 0 0 0 0 1]

**Table 3:** The accuracy of the models set in this paper.

Group Number	Model	Accuracy
Parallel Network		
1	CNN//LSTM×2+Global-Attention	63.284%
	CNN//LSTM×2+Self-Attention	73.031%
2	CNN+Global-Attention//LSTM×2	69.025%
	CNN+Self-Attention//LSTM×2	70.894%
Sequential Network		
3	CNN	70.627%
	CNN+LSTM×2	79.038%
4	CNN+LSTM+Global-Attention+LSTM	71.837%
	CNN+LSTM+Self-Attention+LSTM	71.695%
5	CNN+LSTM×2+Self-Attention	78.772%
	CNN+LSTM×2+Global-Attention	85.427%
6	CNN+Self-Attention	75.033%
	CNN+Global-Attention	58.477%

**Table 4:** The accuracy of the CNN+LSTM×2+global-attention compared to other methods.

	Model	Features	Dataset	Accuracy
Kai Zheng [3]	Multilevel residual CNN	spectrogram	EMO-DB	74.36%
Jianfeng Zhao [6]	2D CNN+LSTM	Log-Mel Spectrogram	EMO-DB	82.42%
Ranjana Dangol [7]	attention-based 3D CNN and LSTM	MFCC	EMO-DB	83.38%
Abdul Malik Badshah [24]	CNN	spectrograms	EMO-DB	73.57%
Mingyi Chen [25]	3-D ACRNN	MFCC	EMO-DB	82.82%
	CNN+LSTM×2+Global-Attention	MFCC	EMO-DB	85.427%

## Data Availability

http://emodb.bilderbar.info/docu/ (accessed on 30 July 2021).

## References

[1] Jeong J., Yang J., Baltes J. (2020). Robot magic show: Human-robot interaction. Knowl. Eng. Rev..

[2] Issa D., Demirci M.F., Yazici A. (2020). Speech emotion recognition with deep convolutional neural networks. Biomed. Signal Process. Control.

[3] Zheng K., Xia Z., Zhang Y., Xu X. (2020). Speech emotion recognition based on multi-level residual convolutional neural networks. Eng. Lett..

[4] Duan C. (2018). A comparative analysis of traditional emotion classification method and deep learning based emotion classification method. Softw. Guide.

[5] Anvarjon T., Mustaqeem, Kwon S. (2020). Deep-Net: A Lightweight CNN-Based Speech Emotion Recognition System Using Deep Frequency Features. Sensors.

[6] Zhao J., Mao X., Chen L. (2019). Speech emotion recognition using deep 1D & 2D CNN LSTM networks. Biomed. Signal Process. Control.

[7] Dangol R., Alsadoon A., Prasad P.W.C., Seher I., Alsadoon O.H. (2020). Speech Emotion Recognition UsingConvolutional Neural Network and Long-Short TermMemory. Multimed. Tools Appl..

[8] Farooq M., Hussain F., Baloch N.K., Raja F.R., Yu H., Bin Zikria Y. (2020). Impact of Feature Selection Algorithm on Speech Emotion Recognition Using Deep Convolutional Neural Network. Sensors.

[9] Zhu L., Chen L., Zhao D., Zhou J., Zhang W. (2017). Emotion Recognition from Chinese Speech for Smart Affective Services Using a Combination of SVM and DBN. Sensors.

[10] Mu Y., Gómez L.A.H., Montes A.C., Martínez C.A., Wang X., Gao H. Speech emotion recognition using convolutional-recurrent neural networks with attention model. Proceedings of the 2017 2nd International Conference on Computer Engineering, Information Science and Internet Technology (CII 2017).

[11] Bahdanau D., Cho K., Bengio Y. (2014). Neural Machine Translation by Jointly Learning to Align and Translate. arXiv.

[12] Zeiler M.D., Fergus R. (2014). Visualizing and understanding convolutional networks. Proceedings of the European Conference on Computer Vision (ECCV).

[13] Wang Z.-Q., Tashev I. Learning utterance-level representations for speech emotion and age/gender recognition using deep neural networks. Proceedings of the 2017 IEEE International Conference on Acoustics, Speech and Signal Processing (ICASSP).

[14] Zhao Z., Zheng Y., Zhang Z., Wang H., Zhao Y., Li C. Exploring Spatio-Temporal Representations by Integrating Attention-based Bidirectional-LSTM-RNNs and FCNs for Speech Emotion Recognition. Proceedings of the Interspeech 2018.

[15] Satt A., Rozenberg S., Hoory R. Efficient Emotion Recognition from Speech Using Deep Learning on Spectrograms. Proceedings of the Interspeech 2017.

[16] Cummins N., Amiriparian S., Hagerer G., Batliner A., Steidl S., Schuller B.W. An Image-based Deep Spectrum Feature Representation for the Recognition of Emotional Speech. Proceedings of the 25th ACM International Conference on Multimedia.

[17] Lee J., Tashev I. High-level feature representation using recurrent neural network for speech emotion recognition. Proceedings of the Interspeech 2015.

[18] Guo L., Wang L., Dang J., Zhang L., Guan H. A Feature Fusion Method Based on Extreme Learning Machine for Speech Emotion Recognition. Proceedings of the 2018 IEEE International Conference on Acoustics, Speech and Signal Processing (ICASSP).

[19] Pascanu R., Gulcehre C., Cho K., Bengio Y. (2013). How to construct deep recurrent neural networks. arXiv.

[20] Hochreiter S., Schmidhuber J. (1997). Long short-term memory. Neural Comput..

[21] Du Q., Gu W., Zhang L., Huang S.L. Attention-based LSTM-CNNs For Time-series Classification. Proceedings of the 16th ACM Conference on Embedded Networked Sensor Systems.

[22] Scherer K. (2003). Vocal communication of emotion: A review of research paradigms. Speech Commun..

[23] Burkhardt F., Paeschke A., Rolfes M., Sendlmeier W.F., Weiss B. A database of German emotional speech. Proceedings of the Interspeech—Eurospeech, 9th European Conference on Speech Communication and Technology.

[24] Badshah A.M., Rahim N., Ullah N., Ahmad J., Muhammad K., Lee M.Y., Kwon S., Baik S.W. (2019). Deep features-based speech emotion recognition for smart affective services. Multimed. Tools Appl..

[25] Chen M., He X., Jing Y., Han Z. (2018). 3-d convolutional recurrent neural networks with attention model for speech emotion recognition. IEEE Signal Process. Lett..

